# Posttraumatic Epidermoid Inclusion Cyst Following Untreated Orbital‐Zygomaticomaxillary Fracture: Case Report

**DOI:** 10.1002/ccr3.70473

**Published:** 2025-04-25

**Authors:** David Kiwango Deoglas, Paulo Joseph Laizer, Shaban Daudi Shaban

**Affiliations:** ^1^ Department of Oral and Maxillofacial Surgery School of Dentistry, Muhimbili University of Health and Allied Sciences Dar es Salaam Tanzania; ^2^ Department of Dental Services Muhimbili National Hospital Dar es Salaam Tanzania

**Keywords:** epidermoid cyst, epidermoid inclusion cyst, orbital‐zygomaticomaxillary fracture, posttraumatic cyst

## Abstract

Timely and comprehensive treatment of craniofacial injuries is essential, as complications may arise, including epidermoid inclusion cysts. We hereby present a case of an orbital epidermoid cyst that developed a year after a patient was involved in a motor vehicle accident and sustained facial injuries that remained untreated.

## 1 Introduction

Epidermoid cysts are benign lesions characterized by cystic spaces lined by simple squamous epithelium (epidermoid cyst), containing skin adnexa (“true” dermoid cyst) or tissues of all three germ layers (teratoid cyst) [1]. Head and neck constitute approximately 7% of all cases of epidermoid and dermoid cysts [2]. They can be either primary (congenital) or secondary cysts. Primary lesions are choristomas that involve displacement of epithelial elements during closure of the neural groove or other epithelial fusion lines. Secondary epidermoid cysts result from posttraumatic implantation of surface epithelium into the dermis [2, 3, 4]. Epidermoid inclusion cysts present as a firm, slow‐growing, mobile, painless mass or lump underneath the skin at the subcutaneous‐dermal level, with an intact skin surface with no apparent drainage point. They contain soft, cheesy‐like skin secretions [5]. Epidermoid cysts can be part of features of syndromes like Gardner syndrome, basal‐cell nevus syndrome, and pachyonychia congenita which are associated with facial swellings. Chin, lip, scalp, and cheek have been the common head and neck areas affected by epidermoid cysts [6, 7, 8, 9]. Posttraumatic epidermoid inclusion cysts are rare and occur mainly in the fingers, palms, and soles [10]. As surgery is traumatic, there have been reports of postsurgical epidermoid cysts involving the head and neck region [4, 8].

We present a rare case of posttraumatic epidermoid inclusion cyst of the facial region following an untreated orbital zygomaticomaxillary complex fracture.

## 2 Case History/Examination

A 32‐year‐old male patient presented to the Oral and Maxillofacial Surgery Department at Muhimbili University of Health and Allied Sciences (MUHAS) with complaints of swelling around his right eye for the past year. The patient reported that the swelling was slow‐growing, painless, and not associated with any disturbances in vision or breathing. He noted that the swellings appeared approximately 1 year after sustaining facial injuries from a motor vehicle accident. However, he did not seek treatment for the facial injuries at the time.

**FIGURE 1:**
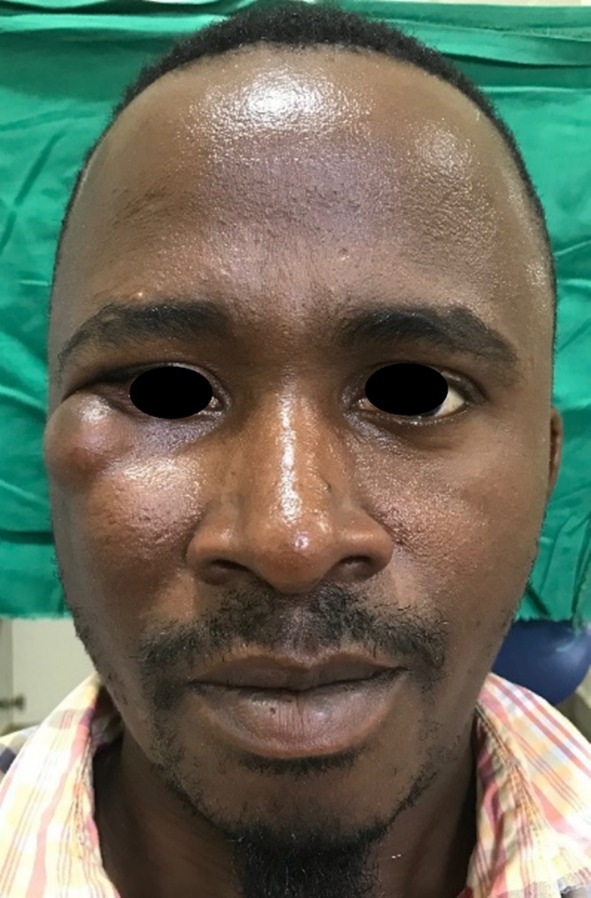
Preoperative; two swelling at the orbital‐zygomaticomaxillary area, intact skin.

Upon examination, two swellings were observed: A 3 × 2 cm swelling at the lower lateral aspect of the right eye and a 4 × 3 cm swelling lateral to the right nasolabial groove (Figure 1).

**FIGURE 2:**
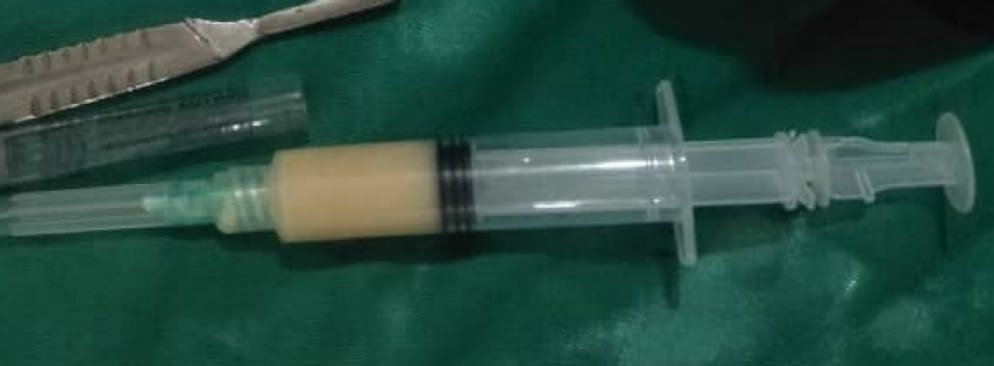
Aspirant via intraoral on the infraorbital cyst, see the cheesy like yellowish secretions.

**FIGURE 3:**
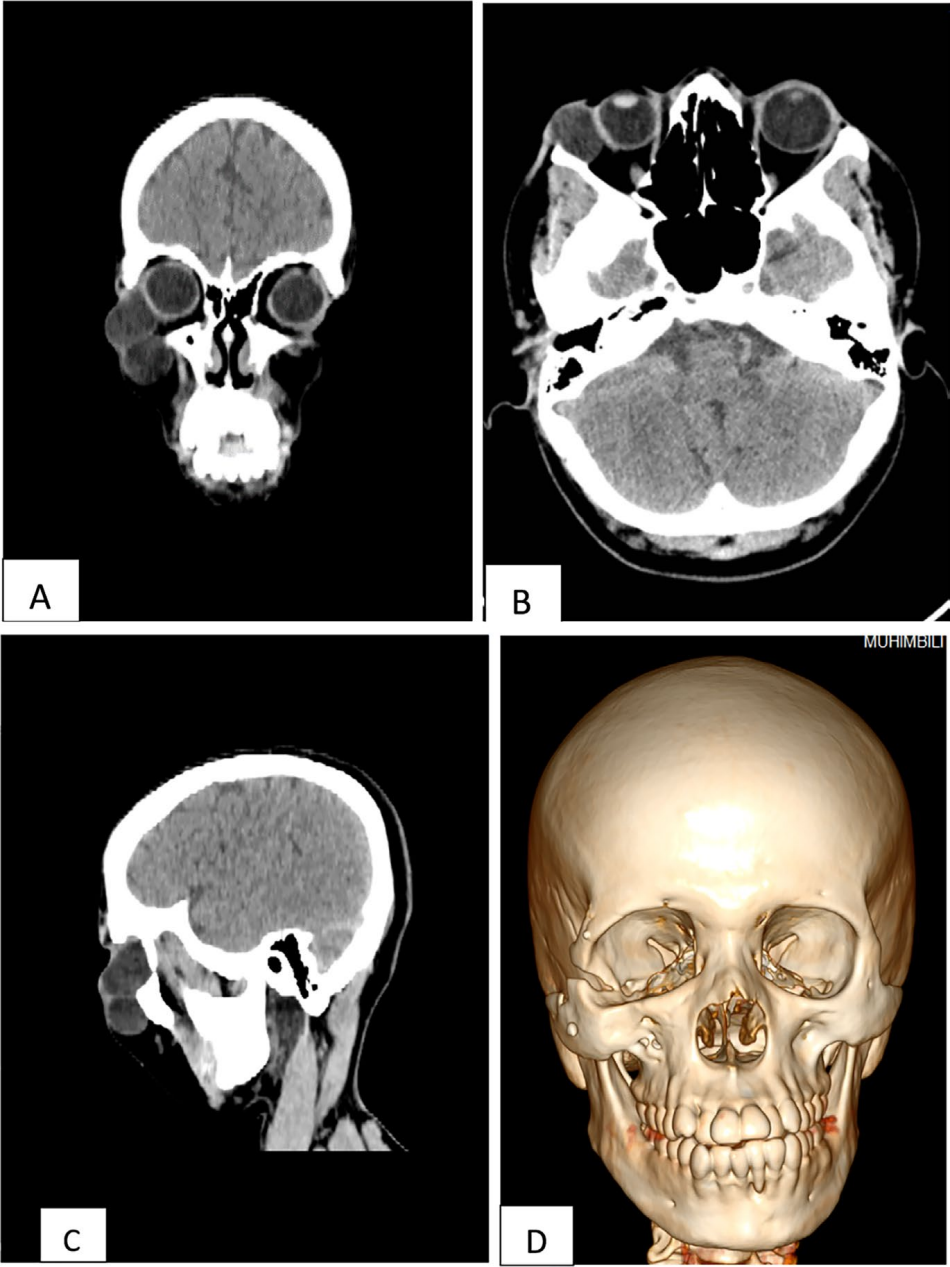
CT scan views (A–C), coronal, axial, sagittal cuts showing the cysts on the orbit and infraorbital regions. (D) A 3D reconstruction CT showing the non‐union lateral orbital rim fracture, see the increased orbital volume on the right orbit signifying a displaced zygomaticomaxillary fracture.

The swellings were fluctuant, mobile, non‐tender, with normal overlying skin. He had intact visual acuity, and eye movement was normal, with no diplopia. Aspiration was done on the inferior cyst via intraoral route, revealing a cheesy‐white granular aspirate (Figure 2). It was submitted for cytological investigation. A CT scan revealed a cystic lesion extending from the lateral side of the globe through a fracture line at the lateral orbital wall to the temporal fossa. Another 4 × 3 cm cystic lesion was below the inferior orbital rim. A 1 cm bone discontinuity was seen at the lateral orbital wall, signifying a non‐union of an inferiorly displaced zygomaticomaxillary complex fracture. Despite the obvious increase in the right orbital volume, there was not a visual disturbance reported prior to the development of the cyst (Figure 3).

## 3 Differential Diagnosis

Dermoid cyst, lipoma, neuroma, neurofibroma, abscess.

## 4 Conclusion and Results

**FIGURE 4:**
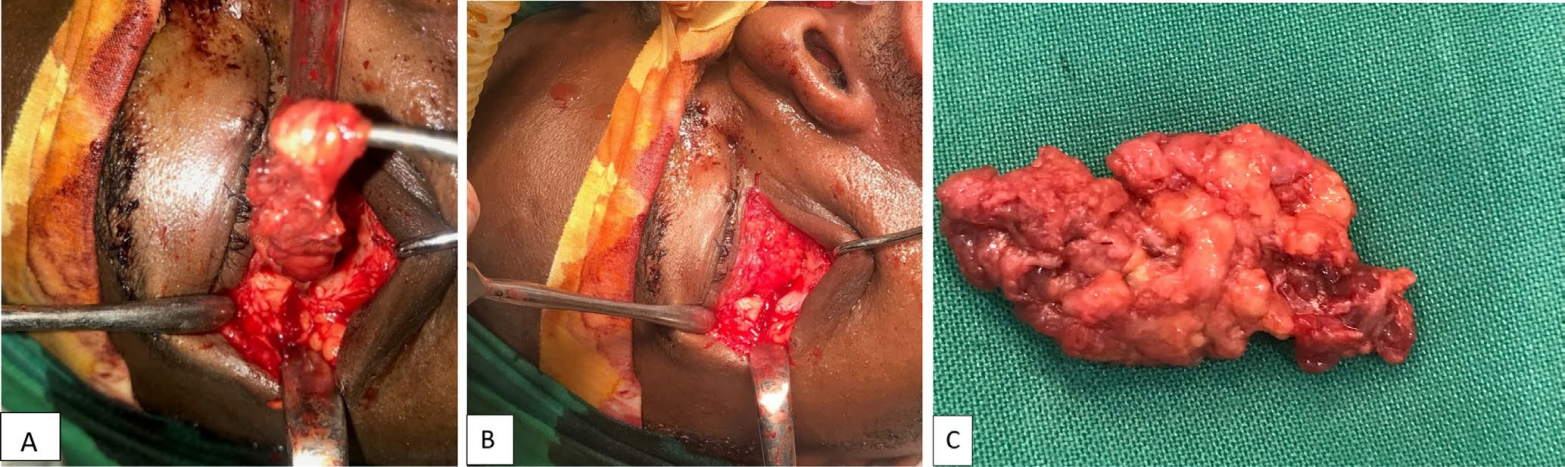
(A, B) Intraoperative; showing the cyst and the fracture area on the lateral orbital rim. (C) The specimen from infraorbital region, excised from the intraoral route, see the fibrotic appearance from the previous FNA procedure.

**FIGURE 5:**
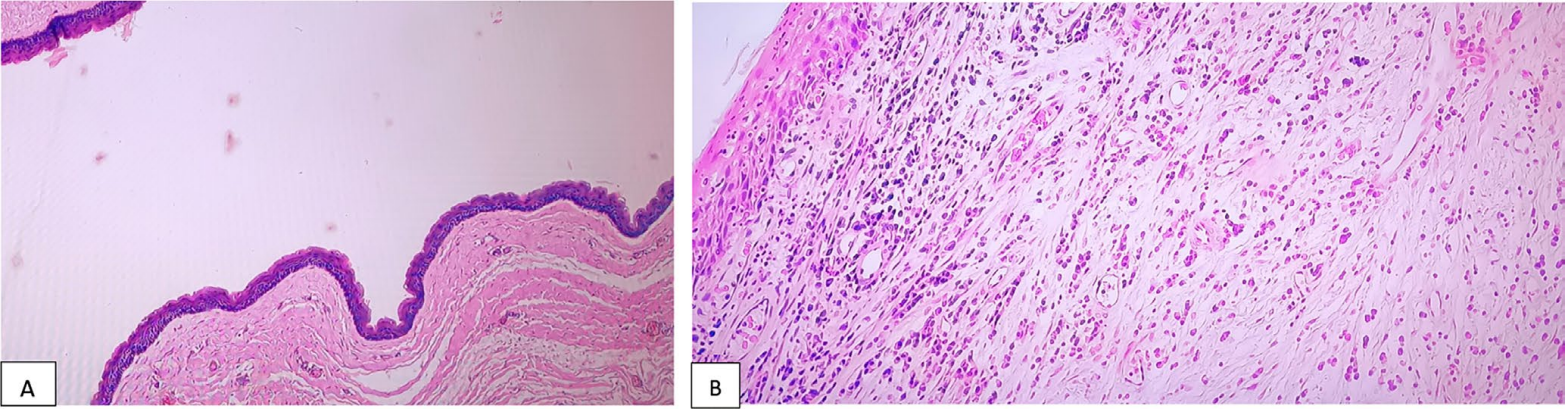
(A) Sections showing a cystic lesion lined by stratified squamous epithelium with a granular layer. Cyst wall does not contain eccrine glands, sebaceous glands or hair follicles. Keratin flakes are not seen in the lumen since the specimen was submitted open. H&E, ×100 magnification. (B) Sections showing a fibrous cyst wall with marked lymphoplasmacytic infiltrates and foreign body type of giant cells (foreign body reaction due to ruptured cysts), H&E ×400 magnification. These features are consistent with epidermal inclusion cyst.

**FIGURE 6:**
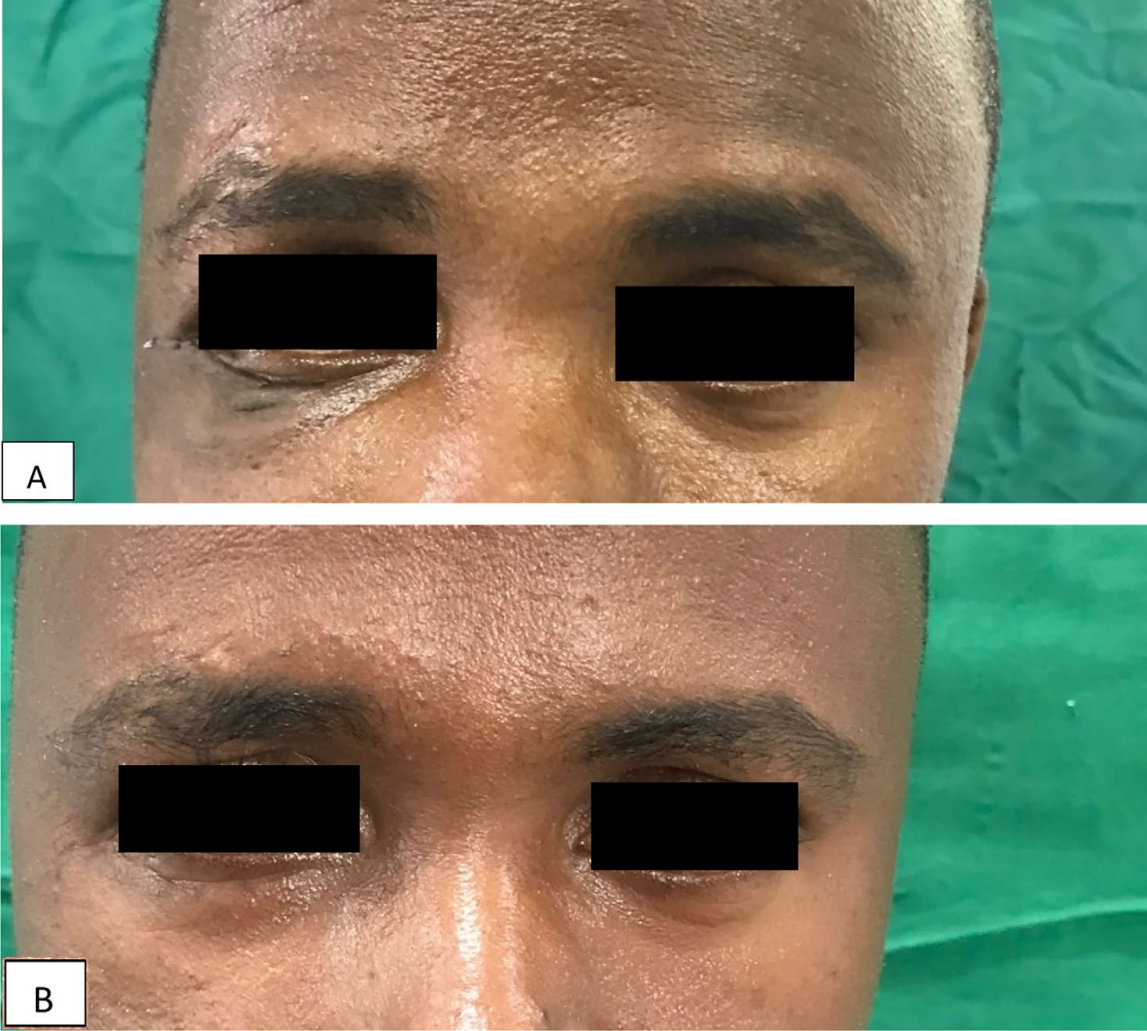
(A, B) Post operative view a, 7 days post operative b, 1 year post‐operative.

**FIGURE 7:**
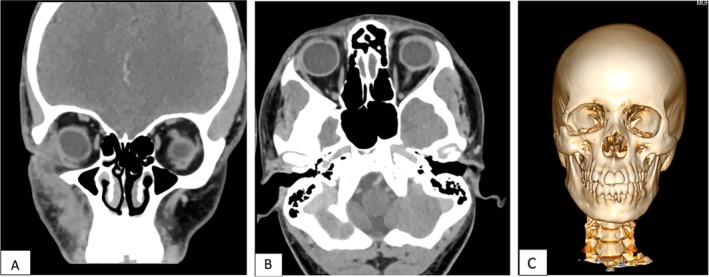
(A, B) 1‐year post‐operative CT scan, coronal and axial views showing no signs of recurrence. (C) CT scan 3D reconstructed view showing the lateral orbital rim defect that was unrepaired.

Surgical excision was planned, the patient was prepared, after obtaining an informed consent after explaining in detail about the surgical procedure and advantages and disadvantages of treatment. An infraorbital incision was used as a surgical access for the orbital cyst and an intraoral vestibular incision for the zygomaticomaxillary cyst. The orbital cyst was found to be firmly adhering to the periosteum at the lateral orbital wall connecting to the zygomatic cyst through the non‐union gap at the lateral orbital rim. The cysts were excised with the involved fibrotic tissues along the non‐union fracture areas (Figure 4). No plates or implants were placed on the lateral orbital wall. Soft tissue repair was done, and the operation ended uneventfully. The specimen was sent for histological evaluation, revealing sections showing a cystic lesion lined by stratified squamous epithelium with a granular layer that did not contain eccrine glands, sebaceous glands, or hair follicles. Keratin flakes were not seen in the lumen since the specimen was submitted open with sections showing a fibrous cyst wall with marked lymphoplasmacytic infiltrates and foreign body type of giant cells that were conclusive of epidermoid inclusion cyst (Figure 5). The patient was followed up, and after 1 year, no ocular or nasal symptoms were reported. There were no signs of recurrence as seen on Figure 6 and on the follow‐up CT scan (Figure 7).

## 5 Discussion

Epidermoid cyst from localized trauma in the craniofacial region has been reported in several literature. After trauma, the skin epithelial cells are driven deep into the dermis and other tissues, where they continue to proliferate and form an epidermoid inclusion cyst [2, 6, 11, 12, 13]. They have been reported in lips, oral mucosa, scalp, and palms, which are common areas to be traumatized [8, 10, 11]. Epidermoid cysts grow slowly and, when in the orbital region, can present with restricted eye movement, double vision, proptosis, and decreased visual acuity. Depending on the location, the cyst can grow to a bigger size associated with pain and facial disfigurements [14]. The epidermoid inclusion cyst can mimic other facial swellings like the findings from Choi et al. [7] where the swelling resembled parotid pleomorphic adenoma. Rupture of the cyst releases keratinaceous contents, which may incite an inflammatory foreign body reaction that causes pain and could be mistaken for infection, as reported by Jeyaraj et al. [9] where the chin mass resembled an infection.

In our case the patient had a right zygomaticomaxillary complex fracture with an inferior displacement following a motor traffic accident. The fractures were untreated leading to a non‐union gap at the lateral orbital wall. An epidermoid cyst developed possibly from the trapped epithelial cells around the orbital region from the initial facial trauma. The swellings grew slowly, were painless without causing much of the patient's concerns until they became big enough to cause aesthetic issues.

FNAC has been used for diagnosis although histology is mandatory to differentiate epidermoid cysts from other cysts. Depending on the location, epidermoid cysts should be distinguished from other cysts including dermoid cyst, lipoma, pilar cyst, furuncle, pilonidal cyst, and steatocystoma. In radiological investigation, CT scan allows for the good assessment of both bone involvement and extensions, with MRI bringing an edge when the cysts have cranial extensions [15].

Surgery is the treatment of choice for epidermoid inclusion, with complete excision being mandatory to avoid recurrence [16, 17]. In this case, the two cysts were found to be interconnected along the fractured lateral orbital rim. The cysts could not be removed without rupturing, as they were adhering to the periosteum and entangled with the fibrotic tissues. There have been reports of possible malignant transformation to squamous cell carcinoma from the residual keratin‐producing lining of these cysts [17, 18]. This warrants meticulous, timely complete surgical excision of the cyst and thorough histopathologic analysis.

Even though posttraumatic epidermoid inclusion cysts in the craniofacial region are relatively rare, they should be considered as one of the differential diagnoses for craniofacial cysts. Treatment of facial fractures and better surgical techniques are of paramount importance to prevent these complications. Although 1 year follow‐up revealed no recurrency, longer follow‐up is required to monitor for recurrence and other outcomes as the healed fractured bones were not corrected in this case.

## Author Contributions

**David Kiwango Deoglas:** conceptualization, methodology, writing – original draft, writing – review and editing. **Paulo Joseph Laizer:** methodology, writing – review and editing. **Shaban Daudi Shaban:** methodology, supervision, writing – review and editing.

## Ethics Statement

This case report was conducted in accordance with the principles of the Declaration of Helsinki. All efforts were made to maintain the patient's privacy and confidentiality.

## Consent

Written informed consent was obtained from the patient for publication of this article and accompanying images.

## Conflicts of Interest

The authors declare no conflicts of interest.

## Data Availability

The authors have nothing to report.

## References

[1] M. Dutta , J. Saha , G. Biswas , S. Chattopadhyay , I. Sen , and R. Sinha , “Epidermoid Cysts in Head and Neck: Our Experiences, With Review of Literature,” Indian Journal of Otolaryngology and Head & Neck Surgery 65, no. SUPPL 1 (2013): 14–21. 24427609 10.1007/s12070-011-0363-y PMC3718960

[2] C. E. Noffke , “Implantation‐Type Epidermoid Cyst of the Mandible,” Dentomaxillofacial Radiology 28, no. 6 (1999): 383–385. 10578196 10.1038/sj/dmfr/4600477

[3] G. Tchernev , I. Temelkova , I. Yungareva , and U. Wollina , “Multiple Epidermal Cysts of the Scalp: Dermatosurgical Approach With Favourable Outcome!,” Open Access Macedonian Journal of Medical Sciences 7, no. 9 (2019): 1509–1511. 31198464 10.3889/oamjms.2019.257 PMC6542396

[4] S. K. Mandal , A. Mandal , and A. A. Bandyopadhy , “Post Surgical Giant Epidermal Inclusion Cyst of the Lid and Orbit‐ A Rare Case,” Journal of Clinical and Diagnostic Research 9, no. 9 (2015): ND01. 10.7860/JCDR/2015/13858.6573 PMC4606261 26500932

[5] T. O. Acarturk and G. M. Stofman , “Posttraumatic Epidermal Inclusion Cyst of the Deep Infratemporal Fossa,” Annals of Plastic Surgery 46, no. 1 (2001): 68–71, 10.1097/00000637-200101000-00015. 11192040

[6] W. C. Wang , L. M. Lin , Y. H. Shen , Y. J. Lin , and Y. K. Chen , “Concurrent Extravasation Mucocele and Epidermoid Cyst of the Lower Lip: A Case Report,” Kaohsiung Journal of Medical Sciences 21, no. 10 (2005): 475–479. 16302452 10.1016/S1607-551X(09)70154-8 PMC11918009

[7] H. J. Choi , J. H. Lee , and Y. M. Lee , “Traumatic Epidermoid Inclusion Cyst on Cheek Area,” Journal of Craniofacial Surgery 27, no. 4 (2016): e343–e344. 27244199 10.1097/SCS.0000000000002509

[8] N. Swain , A. U. Bhandarwar , S. Patel , J. Pathak , and A. Gandevivala , “Postsurgical Epidermal Inclusion Cyst in the Cheek Region,” Journal of Contemporary Dentistry 7, no. 3 (2017): 178–180.

[9] P. Jeyaraj and N. K. Sahoo , “An Unusual Case of a Recurrent Seborrheic/Epidermal Inclusion Cyst of the Maxillofacial Region,” Journal of Oral and Maxillofacial Surgery 14 (2015): 176–185. 10.1007/s12663-012-0408-0 PMC4379255 25838695

[10] J. E. Choi , I. H. Kwon , S. H. Seo , Y. C. Kye , and H. H. Ahn , “Pathogenesis of Plantar Epidermal Cyst: Three‐Dimensional Reconstruction Analysis,” Annals of Dermatology 28, no. 1 (2016): 133–135, 10.5021/ad.2016.28.1.133. 26848239 PMC4737825

[11] S. Samdani , G. S. Kalra , and D. S. Rawat , “Posttraumatic Intradiploic Epidermoid Cyst of Frontal Bone,” Journal of Craniofacial Surgery 24, no. 2 (2013): e128–e130, 10.1097/SCS.0b013e3182700a03. 23524808

[12] F. Zhang , Y. N. Xu , W. Zhao , Y. H. Wang , and J. Q. He , “Posttraumatic Giant Intradiploic Epidermoid Cyst of Orbital Roof,” Journal of Craniofacial Surgery 32, no. 1 (2021): E102–E103. 32675762 10.1097/SCS.0000000000006762

[13] F. W. G. Costa , F. S. R. Carvalho , F. N. Chaves , et al., “Epidermoidna cista nastala na bukalnoj sluznici: Prikaz slučaja i pregled literature,” Acta Stomatologica Croatica 49, no. 1 (2015): 65–73.27688388

[14] D. Veselinović , D. Krasić , I. Stefanović , et al., “Orbital Dermoid and Epidermoid Cysts: Case Study,” Srpski arhiv za celokupno lekarstvo 138, no. 11–12 (2010): 755–759. 21361151 10.2298/sarh1012755v

[15] J. H. Cho , T. Y. Jung , I. Y. Kim , S. Jung , S. S. Kang , and S. H. Kim , “A Giant Intradiploic Epidermoid Cyst With Perforation of the Dura and Brain Parenchymal Involvement,” Clinical Neurology and Neurosurgery 109, no. 4 (2007): 368–373. 17254702 10.1016/j.clineuro.2006.12.011

[16] N. Pushker , R. Meel , A. Kumar , S. Kashyap , S. Sen , and M. S. Bajaj , “Orbital and Periorbital Dermoid/Epidermoid Cyst: A Series of 280 Cases and a Brief Review,” Canadian Journal of Ophthalmology 55, no. 2 (2020): 167–171. 31712044 10.1016/j.jcjo.2019.08.005

[17] A. N. Morritt , N. Tiffin , and T. M. Brotherston , “Squamous Cell Carcinoma Arising in Epidermoid Cysts: Report of Four Cases and Review of the Literature,” Journal of Plastic, Reconstructive and Aesthetic Surgery 65, no. 9 (2012): 1267–1269. 10.1016/j.bjps.2012.02.007 22364661

[18] U. Wollina , D. Langner , G. Tchernev , K. França , and T. Lotti , “Epidermoid Cysts—A Wide Spectrum of Clinical Presentation and Successful Treatment by Surgery: A Retrospective 10‐Year Analysis and Literature Review,” Open Access Macedonian Journal of Medical Sciences 6, no. 1 (2018): 28–30. 29483974 10.3889/oamjms.2018.027 PMC5816307

